# The Consequence of Excessive Consumption of Cow’s Milk: Protein-Losing Enteropathy with Anasarca in the Course of Iron Deficiency Anemia—Case Reports and a Literature Review

**DOI:** 10.3390/nu13030828

**Published:** 2021-03-03

**Authors:** Karolina Graczykowska, Joanna Kaczmarek, Dominika Wilczyńska, Ewa Łoś-Rycharska, Aneta Krogulska

**Affiliations:** 1 SRC Pediatrics, Allergology and Gastroenterology, Ludwik Rydygier Collegium Medicum in Bydgoszcz, Nicolaus Copernicus University, 87-100 Toruń, Poland; asiakaczmarek.j@gmail.com; 2 Department of Pediatrics, Allergology and Gastroenterology, Ludwik Rydygier Collegium Medicum in Bydgoszcz, Nicolaus Copernicus University, 87-100 Toruń, Poland; d.wilczynska@cm.umk.pl (D.W.); ewa.los@cm.umk.pl (E.Ł.-R.); aneta.krogulska@cm.umk.pl (A.K.)

**Keywords:** iron deficiency anemia, protein-losing enteropathy, iron deficiency, edema, cow’s milk protein allergy, cow’s milk, diet, health

## Abstract

Cow’s milk is a key component of a child’s diet. While the consumption of even trace amounts can result in allergy to its proteins and/or hypolactasia, excessive cow’s milk consumption can result in numerous health complications, including iron deficiency, due to the diet being improperly balanced. Although the incidence of iron deficiency has declined, it remains the most widespread nutritional deficiency globally and the most common cause of anemia. One rare consequence of anemia caused by iron deficiency is protein-losing enteropathy; however, the mechanisms of its development are unclear. The following manuscript, based on a literature review, presents two rare cases of children, a 16-month-old boy and a 2.5-year-old girl, who developed severe microcytic anemia, enteropathy with hypoalbuminemia, and anasarca as a result of excessive cow’s milk consumption. It highlights the possible relationship between excessive consumption of cow’s milk in children and severe iron deficiency anemia with accompanying hypoalbuminemia; it may also result in serious clinical conditions, even in children that do not demonstrate food hypersensitivity.

## 1. Introduction

Cow’s milk is often a key component of a child’s diet when consumed in normal amounts; however, while trace amounts can cause the child to develop an allergy to milk proteins and/or hypolactasia, excessive consumption can also result in numerous health complications, perhaps even iron deficiency, due to an improperly balanced diet. However, due to the greater availability of enriched infant formula, the increasing standard of living and the use of iron supplementation in infants who are exclusively breastfed after four months of age, recent years have seen a significant decrease in the incidence of iron deficiency. Nevertheless, it remains the most common dietary deficiency worldwide [1,2,3,4]. 

Iron plays a key role in many biochemical processes, including neurological development, oxygen transport, and energy metabolism [4]. It is needed by almost every cell in the body to function properly. Iron deficiency impairs the functioning of various organs, resulting in hypoxia of the heart muscle (leading to the development of tachycardia, murmurs, and arrhythmia), cerebral hypoxia (resulting in weakness, drowsiness, apathy, irritability, pain and dizziness, tinnitus, spots in front of the eyes, impaired memory and concentration of attention), and decreased immunity (with frequent respiratory tract infections), as well as disorders of the skin and its appendages, and of the mucous membranes [5,6,7]. Iron deficiency also affects the tissues of the digestive tract, resulting in disorders such as glossitis, angular cheilitis, dysphagia, and decreased stomach acidity [7]. 

The most important consequence of iron deficiency is iron deficiency anemia (IDA). It is also the most common cause of anemia, its incidence peaking in young children, and again in teenage girls who have started their periods [5]. 

The prevalence of IDA was found to be 1.1% among children aged one to five months and 2.7% among those aged one to two years in the USA [8], and to range from 2% to 4% among all children in Europe [9]. IDA appears to be more common in young boys (3.1%) than girls (1.3%) [10], and is significantly more common in developing countries than in developed ones [2]. Worldwide, about 293 million preschool children are estimated to have anemia, of which about 50% of cases are due to iron deficiency [11]. The prevalence of IDA remains high, especially among poor families [9]. Despite these high figures, it is important to emphasize that IDA is one of the most significant modifiable risk factors for premature death [12]. 

One highly significant risk factor for IDA is the consumption of large amounts of cow’s milk without iron supplementation [13], which has also been associated with occult gastrointestinal bleeding [2]. In addition, recent studies have shown that children breastfed for more than six months are also at a higher risk of iron deficiency, and that naturally-fed infants are more likely to demonstrate IDA than those given formula milk alone or a mixture of breast and formula milk [3]. Interestingly, exclusively breastfed babies have been found to receive less iron with their food than those receiving formula alone or mixed formula [14]. 

IDA is most often manifested in children as fatigue, weakness, tachycardia, and irritability, as well as pale skin and mucous membranes, and less commonly as pica and weakened nails and hair [5,6,15]. In infants and young children, long-term IDA can lead to impaired psychomotor and mental development [5,6]. Furthermore, iron deficiency occurring early in life, i.e., after birth, can cause structural changes in the brain: Iron deficiency occurring in the first months after birth has been associated with microstructural damage to the brain tissue in pigs; more worrying, this damage appears irreversible, even when correct iron levels are restored [16]. 

In cases where iron levels are significantly reduced, it is possible that protein-losing enteropathy (PLE) can occur [1]. This condition is a relatively rare complication of IDA consisting of the abnormal loss of serum proteins through the gastrointestinal tract, leading to hypoproteinemia and edema [1,17]. Of all the proteins in the body, the most susceptible to depletion is albumin: While only 10% of total albumin is lost through the gastrointestinal tract under physiological conditions, this percentage may rise to 60% in the course of PLE. Although albumin production also increases, it cannot compensate for such high losses [17]. It was first proposed that excessive loss of albumin may occur due to damage to the gastrointestinal mucosa in the course of Ménétrier’s disease in 1947 [18]. 

The causes of PLE can be divided into two types: Those resulting from damage to the mucous membranes and those related to lymphatic abnormalities in the gastrointestinal tract. Damage to the gastrointestinal mucosa may occur in the course of inflammatory/ulcerative diseases of the gastrointestinal tract and non-ulcerative diseases/non-gut conditions. In addition, disturbances in the functioning of the lymphatic system may be primary (hereditary intestinal lymphangiectasia) or secondary [17,18,19,20]. In the case of secondary disorders, it is essential to treat the underlying diseases [17]. The causes of PLE are summarized in Table 1.

Severe IDA can result in PLE, and rare cases have been described of the co-occurrence of PLE, hypoalbuminemia, and anasarca edema with IDA. In children, this condition is solely caused by excessive consumption of cow’s milk; however, due to the recent widespread prevention of iron deficiency and the decline in the incidence of severe IDA [1,21,22] such cases are very rare [6]. 

We present two cases of children with enteropathy, edema, hypoalbuminemia, and severe life-threatening IDA associated with excessive consumption of cow’s milk. 

## 2. Case Reports

### 2.1. Case I 

Patient P.P., a 16-month-old boy (pregnancy I, uncomplicated, by the vaginal route, delivery at term, Apgar 9 points, with unburdened perinatal and family history), was admitted to hospital due to edema and suspected malabsorption syndrome.

According to the medical history, the boy had reported edema of the eyelids two weeks earlier, which then became generalized. The parents were also concerned about loose stools: They reported up to four a day, which were periodically mixed with mucus. Until this time, the child had been healthy. The nutritional medical history indicated that the child had been consuming up to one liter of cow’s milk per day, starting from the age of six months; this was due to family preferences. Although solids were introduced, they were consumed with cow’s milk. Appetite deteriorated, with the appearance of edema occurring two weeks before hospitalization.

On admission to the clinic, physical examination revealed pale skin and mucosa, as well as edema localized around the eyelids and lower limbs, accompanied by ascites. The heart rate was 140/min (upper limit of normal). The boy demonstrated a body weight of 12.5 kg, height 86 cm, and a BMI (body mass index) of 16.96 (90 percentile).

The following abnormalities were found in laboratory tests: Significantly decreased hemoglobin (6.7 g/dL) and ferritin (<5 µg/L), decreased mean red blood cell volume (56.3 fL), mean corpuscular hemoglobin (14.9 pg) and mean corpuscular hemoglobin concentration (26.4 g/dL), hypoproteinemia (3.7 g/dL) with hypoalbuminemia (2.0 g/dL), and increased concentration of calprotectin in the feces (1020.6 mg/kg). The peripheral blood smear revealed microcytosis, hypochromasia, prominent anisocytosis, and poikilocytosis. Several urinalyses were normal, with no proteinuria.

Other parameters were within normal ranges, e.g., liver function tests, thyroid function (TSH and fT4), anti-tissue transglutaminase IgA and IgG antibodies, and tests for parasites. The result of the fecal occult blood test was negative. 

Endoscopic examination of the gastrointestinal tract revealed macroscopic signs of gastropathy, as well as a slight swelling of the mucosa in the central part and in the pre-antral area of the stomach. Numerous simple papules were also observed in the duodenum and rectum, and single lumps in the proximal part of the large intestine. In the small intestine, the macroscopic picture of the mucosa was normal, with no signs of villous atrophy. The histopathological examination revealed moderately profuse inflammatory infiltration from lymphocytes with the presence of eosinophils in the gastric and large intestine mucosae (<15 eosinophils/high-power field in histopathological samples taken from different levels of the gastrointestinal tract). 

The concentration of total IgE in serum was also determined: The level was found to be <1.5 IU/mL (normal), with normal results for allergen specific IgE antibodies (asIgE) (<0.15 kU/L for all major allergens, including milk). 

Based on the clinical picture and the results of the conducted tests, protein-losing enteropathy (PLE) in the course of iron deficiency anemia (IDA) was suspected. As the patient had not previously presented any symptoms typical of IgE-mediated or non-IgE-mediated cow’s milk protein allergy (CMPA), and that the family history of allergy was negative, no sensitization to any food allergens was shown, and CMPA was excluded. The other causes of PLE were also ruled out.

The treatment included an intravenous supply of red blood cell concentrate and 20% albumin, together with oral iron supplementation and a dairy-free diet (elemental formula). The boy demonstrated rapid improvement in his clinical condition and normalization of laboratory tests was achieved. The child was discharged home in good condition with a recommendation to continue oral iron supplementation and a dairy-free diet. At discharge, the boy’s weight was 11.5 kg (50–75 percentile).

After two months of treatment, the child’s general condition improved, the symptoms resolved, and physical examination and laboratory tests were normal (total protein 4.6 g/dL, albumin 3.2 g/dL, Hgb 12.1 g/dL). Cow’s milk was gradually included in the diet in recommended amounts, with good tolerance observed for another year. 

### 2.2. Case II

A girl B.C., 2.5 years old (pregnancy II, complicated by gestational diabetes, delivery II, via the vaginal route, on time, assessed at 10 points on the Apgar scale, with an unburdened family history) was admitted to hospital as an outpatient due to generalized edema accompanied by anemia. 

According to the medical history, in the month preceding hospitalization, the patient had regularly passed four liquid stools per day without pathological admixtures; she also reported significantly reduced appetite and lower limb pain. During this period, the girl was consuming more than a liter of cow’s milk each day, as well as porridge prepared with cow’s milk: she refused to eat other products. Complementary products were introduced into the diet as recommended; however, they were only consumed in small amounts, because the patient refused to take them. The girl had been breastfed until six months of age, following which the diet was dominated by modified milk. Cow’s milk was first introduced into the diet one month before hospitalization. Edema of the lower limbs and eyelids had appeared a few days before hospitalization. 

Until the month before hospitalization, the child had been healthy.

On admission, the patient’s condition was defined as average. Physical examination revealed pale skin and mucous membranes, as well as generalized edema. The child’s body weight was 17.5 kg, height 90 cm, and BMI 21.6 (>97 percentile). Her heart rate was 130/min (normal at her age: 76–142/min.)

A number of abnormalities were observed in the laboratory tests: Very low blood hemoglobin level (5.2 g/dL) and significantly decreased iron level (<2 µmol/L), as well as decreased mean red blood cell volume (58.2 fl), mean corpuscular hemoglobin (13.7 pg) and mean corpuscular hemoglobin concentration (23.5 g/dL), hypoproteinemia (4.9 g/dL), and hypoalbuminemia (1.0 g/dL). Stool examinations showed high levels of calprotectin (1553.7 mg/dL) with negative fecal occult blood test and stool cultures. No protein was found in the urinalysis. Other biochemical parameters (e.g., liver function tests, anti-tissue transglutaminase IgA and IgG antibodies) were within normal ranges. Parasitic infections were also excluded.

Endoscopy of the upper and lower gastrointestinal tract was performed. Macroscopically, simple papules were visualized in the antral part of the stomach and the duodenal bulb, as well as in the sigmoid colon and rectum. Histopathological examination of the sigmoid and rectal specimens revealed a slight swelling of the mucosa, diffuse inflammatory infiltrates from lymphocytes, plasmocytes, and eosinophils in the lamina propria (<15 eosinophils/high-power field); however, the gastric mucosa and the rest of the duodenum were found to be normal. 

CMPA was considered in differential diagnosis. The evaluation of asIgE for food allergens confirmed the presence of asIgE to cow’s milk (1.9 kU/L). However, it was decided not to introduce an elimination diet as no clinical symptoms suggesting allergy or food allergy were observed, the child’s and family history were negative, and endoscopy did not reveal characteristic signs of allergy. Additionally, capsule endoscopy suggested that, as far as could be seen, the small and large intestines were normal. CMPA and other causes of PLE were thus excluded.

On the first day of hospitalization, a concentrate of red blood cells and 20% albumin were transfused. It was recommended that oral iron preparations be taken and that the diet should be expanded to include iron-rich foods. 

After a week of hospitalization, the patient’s condition improved significantly, swelling subsided, the stools normalized, and no recurrence of anemia was observed. Laboratory tests showed a slightly decreased hemoglobin level (10.9 g/dL) and a significant decrease in the concentration of calprotectin in stools (134.8 mg/dL). Total protein was decreased (4.9 g/dL), but albumin was normal (3.5 g/dL). The body weight at discharge was 16.5 kg (90–97 centile). The patient was released home in good condition with a recommendation to continue oral iron supplementation and to follow a balanced diet appropriate to her age. The patient’s parents were instructed not to allow their daughter to consume more than 500 mL of milk per day. Further care in a gastroenterology clinic was recommended.

After four months of treatment, at the follow-up visit, the girl remained symptom free; the physical examination and control laboratory tests revealed no abnormalities (Hgb 13.1 g/dL, albumin level 4.7 g/dL). Good tolerance of cow’s milk was observed for another year.

## 3. Discussion

The two described patients demonstrated almost identical clinical pictures, i.e., generalized swelling and loose stools were reported, and testing identified IDA and hypoalbuminemia. The medical history on admission found both the girl and the boy had been consuming considerable amounts of cow’s milk. 

The presented cases confirm previous observations [1,2,6,21,22,23,24,25,26,27,28] suggesting that in young children, excessive consumption of cow’s milk can lead to the development of severe iron deficiency anemia (IDA), followed by protein- losing enteropathy (PLE), hypoalbuminemia and edema. Nevertheless, such cases are very rare. The few cases described so far are summarized in Table 2. 

Such cases, i.e., where PLE arises in the course of IDA resulting from excessive cow milk consumption, have a characteristic clinical picture [1,2,6,21,22,23,24,25,26,27,28], and although the children may be experiencing severe, life-threatening IDA, their parents are more likely to take them to a doctor to address the edema [1,2,6,24]. Importantly, generalized edema may develop in as little as two to three weeks in a previously healthy child [1], as was observed in the presented cases: The edema appeared and then generalized within two weeks in the boy, and within four weeks in the girl.

The medical history indicated that the children had been consuming very large amounts of cow’s milk: Approx. 1 L/day from six months of age by the boy, and for one month before hospitalization by the girl. In addition, neither of the children had previously received iron supplementation. Previous studies have recorded milk consumption levels of 0.7–0.9 L/day [1], and these levels can even exceed 1.9 L/day [2,21]. It is often the only type of food the baby consumes, as noted in the present cases. 

**Figure 1:**
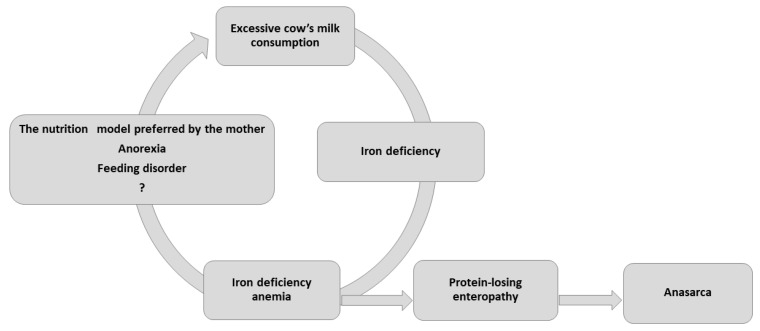
Consequences of excessive cow’s milk consumption.

Although the highest incidence of IDA is typically observed among low socioeconomic groups in developed countries, both children we described were from well-off backgrounds. IDA occurred due to consumption of large amounts of unfortified, pasteurized cow’s milk, compounded by the fact that the children refused to take solids, and consumed little iron-rich food. IDA is known to lead to progressive anorexia and dietary iron deficiency as it worsens, resulting in a potentially fatal vicious cycle (Figure 1).

Stool quality has been inconsistently described in previous reports, and children with PLE can manifest stooling patterns that range from no change from baseline, to diarrhea, and to diarrhea alternating with constipation [6]. Our patients reported passing loose stools with mucus up to several times a day.

In both reported cases, the laboratory tests identified severe microcytic anemia, iron deficiency, and hypoproteinemia with hypoalbuminemia. The hemoglobin values in the described patients were 6.7 and 5.2 g/dL, respectively, mean value 5.9 g/dL. These findings are consistent with previous case reports, where these values ranged between 2 and 10.3 g/dL, mean 5.29 g/dL (Table 2).

The incidence is not related to sex, although there is a slight predominance among girls. Our own material included one boy and one girl. 

Cases of PLE occurring as a result of IDA can be effectively treated by compensating for iron deficiency through oral or intravenous supplementation [1,23,25]. The oral iron treatment typically corrects the hypoproteinemia and clinical edema within one to three weeks [1]. Interestingly, in cases of PLE with IDA, iron supplementation corrects both problems; this was also the case with the children in the present study. In addition, in both cases, the resolution of hypoproteinemia and edema was associated with approximately 1 kg weight loss compared to admission.

The course of the disease in both presented patients was similar, but the management was different. CMPA was not diagnosed in either child. While milk was eliminated from the diet in the boy, normal milk consumption was restored in the girl. Similarly, the literature reports a varied approach to dietary management; nevertheless, introducing treatment with iron preparations results in stabilization of the clinical condition irrespective of the implemented diet, i.e., dairy-free or containing milk. 

As rightly noted by Lundstrom et al., although CMPA may also lead to malabsorption disorders, together with iron deficiency and hypoalbuminemia, in such cases, it would be impossible to achieve remission of symptoms and normalize laboratory test results in children with allergies without simultaneous elimination of cow’s milk proteins [23]. They describe eight cases where edema and hypoalbuminemia disappeared shortly after the introduction of iron preparations, and the hemoglobin and iron values in the blood returned to normal within six weeks. None of the patients was on an elimination diet [23]. Several years later, an analysis of 24 patients with iron deficiency anemia, seven of whom had PLE, found oral iron preparations to demonstrate the same efficacy of treatment, regardless of whether it was combined with a cow’s milk or soymilk diet [25]. 

These observations suggest that the mechanisms underlying PLE and IDA are not associated with hypersensitivity to cow’s milk proteins. In the presented girl, no cow’s milk elimination was applied, yet her clinical condition and laboratory parameters stabilized shortly after iron supplementation. Although the boy was prescribed a dairy-free diet, it should be emphasized that this decision was made to enhance the absorption of amino acids in the small intestine and was not associated with the diagnosis of CMPA as the cause of the symptoms.

It is important to note that non-IgE-mediated CMPA should be considered in the diagnosis of any patient presenting the following symptoms: Prolonged diarrhea, abnormal growth, recurrent vomiting, flatulence, blood in the stools, IDA, or other mild gastrointestinal disorders not responding to standard treatment [29,30]. This diagnosis is strengthened by the presence of skin lesions and/or respiratory symptoms. 

It should also be emphasized that in each case of enteropathy, it is important to consider the possibility of food protein-induced enteropathy (FPE) (e.g., by cow’s milk). Typical symptoms include anorexia and refusal to eat, vomiting, constipation, or diarrhea lasting more than 15 days with or without abnormal growth; these symptoms resolve within four to six weeks after eliminating cow’s milk from the diet. However, in the case of FPE, the symptoms return when milk is reintroduced into the diet. In addition, a diagnosis of FPE should include confirmation by histopathological examination of a fragment of the small intestine: A positive diagnosis requires the presence of damage to the intestinal villi, crypt hyperplasia, and inflammation [29]. In addition, IDA may develop in the course of FPE [13,29,31]. 

Although the clinical picture of the patients described in this publication can resemble that of non-IgE-mediated CMPA, there are significant differences: In the case of allergies, it is impossible to obtain remission of symptoms without the elimination of the allergen, i.e., cow’s milk, and additionally, confirmation of non-IgE-mediated CMPA requires an oral provocation test, in which symptoms reappear after the reintroduction of milk [30]. In PLE and IDA related to excessive consumption of cow’s milk, it is sufficient to introduce iron supplementation and reduce the amount of milk consumed; there is no need to introduce an elimination diet. 

Lai et al. examined 51 patients with IDA, including seven with CMPA [31]. The patients presented with pale skin, anemia, and significant iron deficiency; more than half had hypoalbuminemia and/or a positive fecal occult blood test, and some also demonstrated edema. Importantly, six of the seven CMPA patients were on a diet based on iron-fortified milk, and before the diagnosis of CMPA, they had received oral iron supplementation for one to two months with no obvious improvement in laboratory results. The authors emphasize that insufficient iron supply was excluded as the cause of IDA, and normalization of laboratory test results and symptom resolution could only be achieved by elimination of cow’s milk from the diet [31]. 

**Figure 2:**
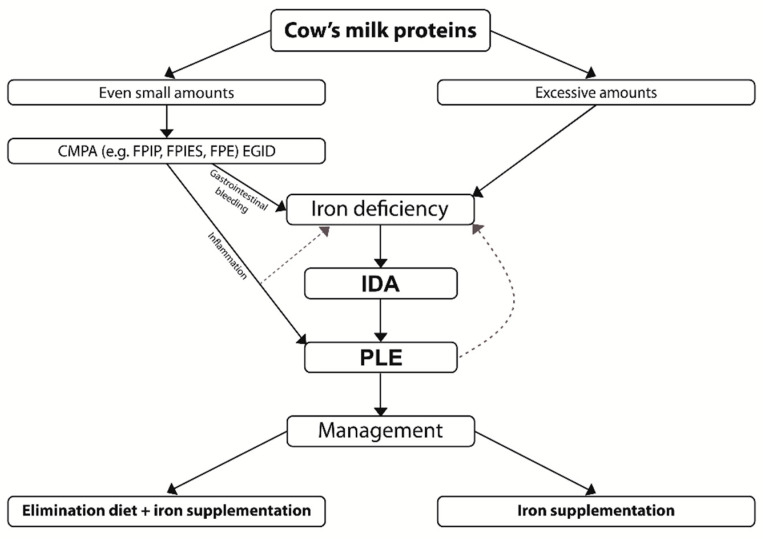
Possible pathways of PLE development through the consumption of varying amounts of cow’s milk proteins (FPIP—food protein-induced proctocolitis, FPIES—food protein-induced enterocolitis syndrome, FPE—food protein-induced enteropathy, EGID—eosinophilic gastrointestinal disorders, IDA—iron deficiency anemia, PLE—protein-losing enteropathy).

It is worth noting that cow’s milk can lead to the development of enteropathy and anemia through two mechanisms (Figure 2):

- In the first, resulting from CMPA, trace amounts of cow’s milk proteins lead to the development of enteropathy through the action of immune disorders, and anemia through bleeding from the gastrointestinal tract;
- The second mechanism is related to severe IDA resulting from excessive consumption of cow’s milk; this in turn causes enteropathy. The mechanism of this disorder has not been fully elucidated [1,23,25]. Recently, it was shown that severe anemia resulted in a disproportionate and persistent increase in intestinal permeability due to the disruption of epithelial adherens junctions, mediated via depletion of E-cadherin (Cdh1) mRNA [32].

Nairz et al. [33] indicate that in patients with inflammation-related diseases (e.g., allergy), immune activation and iron deficiency can lead to anemia due to disruption of iron homeostasis. Although the etiology of anemia of inflammation differs from that of IDA, it is difficult to differentiate these conditions in the typical clinical setting, as both types of anemia exhibit low hemoglobin and low iron status in blood tests. They also propose that a clear link exists between allergic diseases and anemia and/or iron levels [33].

The patients in the present article did not display any symptoms typical of CMPA, or the cause-and-effect relationship typical of food allergy, i.e., a relationship between the disease and the diet used. This indicates that PLE must have formed through the second mechanism given above.

In addition, patients experiencing PLE in the course of IDA associated with excessive cow’s milk consumption do not present changes characteristic of CMPA in intestinal histopathology [23]. For example, they do not demonstrate significant numbers of lymph nodes, moderate or advanced changes to the mucosa, increased numbers of intraepithelial lymphocytes and eosinophils, or IgA—and IgM—containing cells in the lamina propria [13,23]. In fact, patients were found to present mild, nonspecific changes in the intestinal mucosa, and the numbers of IgM—and IgA—containing cells in them were below normal; in contrast, patients with CMPA, even those with mild clinical symptoms, typically demonstrate increased numbers of these cells [23].

Similarly, Tracy et al. report that endoscopic examination did not reveal any changes in their patients with PLE in the course of IDA, and question the value of endoscopic examinations [28]. In our patients, although the endoscopic and histopathological examinations suggested no disturbances within the intestinal villi, they nevertheless identified inflammation, papules, and slight swelling of the mucosa.

Although allergy to cows’ milk proteins was initially recognized as the primary cause of PLE observed in patients with severe IDA, previous studies do not always support this hypothesis. A study of several Finnish children who had developed PLE as a result of excessive consumption of cow’s milk found the introduction of oral iron supplementation to result in normalization of laboratory test results and resolution of clinical symptoms, with or without the elimination of cow’s milk from the diet [23,25]. In these patients, hypersensitivity to cow’s milk may not in itself be the cause of PLE: The main role is played by dietary iron deficiency (including excess cow’s milk).

Due to their rapid growth and dynamic development, infants and young children typically have a high demand for iron. This amount is approximately 11 mg/day in infants aged over six months, and 7 mg/day in children aged one to three years [34]. A diet rich in cow’s milk and low in supplementary iron can lead to deficiency, resulting in inter alia the development of microcytic anemia. As such, complete diet histories should be part of every pediatric visit in the first years of life. In addition, parents should be encouraged to include iron-containing foods in their child’s diet after four months of age and should be warned about the dangers of excessive milk consumption: It is recommended that young children should not consume more than three servings a day of dairy products, with a maximum amount of 2 × 210 mL cow’s milk and one serving of dairy per day. 

Excessive consumption of cow’s milk leads to iron deficiency through several mechanisms.

- Cow’s milk has very low iron content (only 0.5 mg/L). This is similar to milk from other animals: Goat’s and sheep’s milk contains similar amounts as cow’s milk, while mares’ milk has slightly higher levels [35];
- Excessive consumption of cow’s milk can lead to iron deficiency anemia caused by dietary imbalance, decreased consumption of solids, and increased consumption of an iron-deficient milk diet;
- Cow’s milk contains predominantly non-heme iron, which is much less digestible than heme iron [36];
- Due to its high casein and calcium content, cow’s milk prevents the digestive tract from absorbing iron from other foods [36,37]. This applies to non-heme iron, which makes up the majority of dietary iron [37]. Cow’s milk has four times the calcium content of human milk, and this also competes with iron for absorption [13];
- Cow’s milk is poor in vitamin C, and pasteurization additionally reduces its content [13,26]. It is well known that vitamin C increases the absorption of iron from the gastrointestinal tract. It is worth mentioning that although cow’s milk and human breast milk have similar iron contents, human milk demonstrates 2.5 times greater iron bioavailability [26];
- In addition, it has been found that healthy infants physiologically lose small amounts of blood through the gastrointestinal tract [36,37]. Excessive consumption of cow’s milk in young children can increase the rate of blood loss, which further increases the need for iron [38]. Studies indicate that the stools of children with simultaneous PLE and IDA have higher mean hemoglobin content than normal: 1.48 mg of hemoglobin per gram of stools compared to about 0.5–0.8 mg per gram [21]. Although gastrointestinal bleeding was considered a cause of iron loss and subsequent iron deficiency in PLE, its severity appears to be insufficient to cause IDA [23,25].

In both presented patients, a fecal occult blood test was performed in order to exclude gastrointestinal bleeding as the cause of iron deficiency anemia. The result was negative in both cases. 

The limitation of the presented cases is a lack of altha-1-antitripsin evaluation; however, the diagnosis of PLE was based on symptoms, hypoproteinemia, and the exclusion of other causes of hypoproteinemia. 

In pediatrics, the incidence of hypoproteinemia with hypoalbuminemia is most often associated with renal disorders; however, proteinuria is not observed in children with PLE, which was also the case in our patients.

The combination of a normal urine test and negative fecal occult blood test indicates that the observed low levels of protein and albumin are not related to the primary renal or gastrointestinal causes of PLE. Further tests carried out on the two presented children allowed celiac disease, infectious diseases of the gastrointestinal tract, and other causes of malabsorption disorders to be excluded. In addition, CMPA was ruled out by the absence of symptoms typical of food allergy or any other type of allergic disease, improvement after Fe supplementation, and the absence of complaints on a well-balanced dairy diet, without symptoms for another year. Additionally, we would like to emphasize that although the symptoms were severe and life-threatening, they lasted briefly, which is not typical of non-IgE-mediated food allergy. In addition, in case I, after its elimination, milk was gradually introduced, but only within 2 weeks, which is too short period to recognize that the absence of symptoms after its implementation was a result of acquiring tolerance. In both presented children, IDA was diagnosed as a result of nutritional deficiencies caused by excessive milk consumption. 

The mechanisms leading to the development of the described enteropathy/PLE are not fully understood; however, it has been shown that it is not caused by CMPA [1,23,25]. PLE and IDA appear to co-exist mainly in cases of severe iron deficiency and without primary gastrointestinal pathology [23]. Significant iron deficiency is believed to be a factor leading to intestinal barrier dysfunction. This is confirmed by the fact that edema and hypoproteinemia disappeared soon after the introduction of iron supplementation, even before the normalization of red cell parameters, which also rules out celiac disease or CMPA as the cause of PLE [1,23,25]. It is believed that anemia and iron deficiency adversely affect tissue metabolism and contribute to intestinal barrier dysfunction, which results in increased loss of blood plasma proteins [1,27]. It is possible that in these cases, the production of peptidases and amino acid transporters is dysregulated, resulting in both the impaired absorption of amino acids into the blood from the intestinal lumen and the loss of plasma proteins to the intestinal lumen [1].

It is important to note that not all children with severe iron deficiency associated with excessive consumption of fresh cow’s milk will develop PLE [2]. Rather, it seems that a certain threshold of iron deficiency must be crossed before PLE can develop in the course of IDA: Most children with IDA do not develop PLE and do not develop visible edema [1].

## 4. Conclusions

The occurrence of severe iron deficiency anemia (IDA) in children is associated with hypoalbuminemia, and can be caused by excessive consumption of cow’s milk. The mechanism of this disorder is not fully understood, but it appears to occur independently of CMPA. In children with edema and symptoms of iron deficiency anemia, the differential diagnosis should consider enteropathy with protein loss related to excessive consumption of cow’s milk. 

When caring for a small child, it is important to remember the need for a properly balanced diet: cow’s milk should not be the main source of nutrients, and the diet must include enough iron for growth and development. In addition, all pediatric visits in the first years of life should include a thorough nutritional medical history. In addition, pediatricians should be made aware of the link between severe iron deficiency anemia and anasarca edema, particularly since milk is such a fundamental element of a child’s diet and the condition is so rare. Further studies are needed to clarify the role of iron in maintaining barrier function and protein-handling in the gastrointestinal tract.

## Figures and Tables

**Table 1:** Causes of protein-losing enteropathy (PLE) in children [19].

Causes	Examples
Mucosal Injury	
Inflammatory and Ulcerative Diseases	Inflammatory bowel disease: Crohn’s disease/ulcerative colitis Infections Bacterial: Salmonella, Shigella, Campylobacter, *Clostridium difficile* Parasitic: *Giardia lamblia* Viral: Rotavirus Gastrointestinal malignancies: Esophageal, gastric, colonic adenocarcinomas Lymphoma Kaposi’s sarcoma NSAID enteropathy Graft vs. host disease Necrotizing enterocolitis Ulcerative ileitis
Non-Ulcerative Diseases	Hypertrophic gastropathies (Ménétrier’s disease) Eosinophilic gastroenteritis Food-induced enteropathy Celiac disease Tropical sprue Small intestinal bacterial overgrowth Vasculitic disorders: SLE, HSP
Lymphatic Abnormalities	
Primary Intestinal Lymphangiectasia (PIL)	
Secondary Intestinal Lymphangiectasia	Obstructive: Crohn, sarcoidosis, lymphoma Elevated lymph pressure: congestive heart failure, constrictive pericarditis Syndromal: Turner, Noonan, Hennekam, Klippel–Trenaunay, von Recklinghausen after Fontan procedure

**Table 2:** Summary of published case reports of iron deficiency anemia (IDA) and PLE associated with excessive cow’s milk intake.

References	Number of Cases Child Age	Symptoms	Amount of Cow’s Milk Per Day	Hemoglobin g/dL	Total Protein g/dL	Albumin g/dL	Cow’s Milk Elimination
Lundstrom et al. [23] 1983	8 patients: 8–24 months	edema	no data	2.0–7.6	3–4.8	no data	no
Hamrick [22] 1994	19 months	pallor, edema (facial, periorbital, extremities)	>830 mL	4.2	3.0	1.8	yes
Bondi et al. [26] 2009	19 months 24 months	pallor mild fever, edema (feet, knee), pica (soil)	1400 mL 1800 mL	2.3 10.3	no data	no data	no data
Vogelaar et al. [6] 2014	13 months	edema (periorbital, abdominal, bilateral lower extremity), alternating diarrhea and constipation	1300–1600 mL	7	2.7	1.4	no (only minimized intake)
Yasuda et al. [1] 2018	20 months	pallor, edema (periorbital, bilateral lower extremity, abdominal), diarrhea (5–6 loose to watery stools per day)	>800 mL	6.4	3.0	1.6	yes
Mantadakis et al. [2] 2018	13 months	pallor, edema (periorbital, bilateral lower extremity), fatigue, irritability	>1200 mL	3.8	no data	2	no (only minimized intake)
Carbonell et al. [27] 2019	22 months	pallor, edema (periorbital, lower extremities), fever, dark stool, atopic dermatitis	480–720 mL	5.1	no data	2.1	yes
Kamzan et al. [24] 2020	15 months	pallor, edema (periorbital)	>900 mL	3.9	3.3	1.9	no (only minimized intake)
Tracy et al. [28] 2020	7 patients: 14–27 months	edema (periorbital), abdominal distension, pallor	730–2120 mL	4–8	no data	1.5–2.5	yes
